# Association of Insulin Resistance and β-cell Function With Bone Turnover Biomarkers in Dysglycemia Patients

**DOI:** 10.3389/fendo.2021.554604

**Published:** 2021-03-25

**Authors:** Hui Guo, Chiyu Wang, Boren Jiang, Shaohong Ge, Jian Cai, Ying Zhou, Rong Ying, Kexi Zha, Ji Zhou, Ningjian Wang, Chunfang Zhu, Chenyu Cao, Liqin Zhang, Tao Gu, Yan Zhao, Yingli Lu, Zengmei An

**Affiliations:** ^1^Institute and Department of Endocrinology and Metabolism, Huangpu Branch of Shanghai Ninth People’s Hospital, Shanghai, China; ^2^Institute and Department of Endocrinology and Metabolism, Shanghai Ninth People’s Hospital, Shanghai Jiao Tong University School of Medicine, Shanghai, China

**Keywords:** insulin resistance, β-cell function, homeostatic model assessment, turnovers, β-CTX, P1NP, osteocalcin

## Abstract

**Background:**

The interrelation between glucose and bone metabolism is complex and has not been fully revealed. This study aimed to investigate the association between insulin resistance, β-cell function and bone turnover biomarker levels among participants with abnormal glycometabolism.

**Methods:**

A total of 5277 subjects were involved through a cross-sectional study (METAL study, http://www.chictr.org.cn, ChiCTR1800017573) in Shanghai, China. Homeostasis model assessment of insulin resistance (HOMA-IR) and β-cell dysfunction (HOMA-%β) were applied to elucidate the nexus between β-C-terminal telopeptide (β-CTX), intact N-terminal propeptide of type I collagen (P1NP) and osteocalcin (OC). β-CTX, OC and P1NP were detected by chemiluminescence.

**Results:**

HOMA-IR was negatively associated with β-CTX, P1NP and OC (regression coefficient (β) -0.044 (-0.053, -0.035), Q4vsQ1; β -7.340 (-9.130, -5.550), Q4vsQ1 and β -2.885 (-3.357, -2.412), Q4vsQ1, respectively, all P for trend <0.001). HOMA-%β was positively associated with β-CTX, P1NP and OC (β 0.022 (0.014, 0.031), Q4vsQ1; β 6.951 (5.300, 8.602), Q4vsQ1 and β 1.361 (0.921, 1.800), Q4vsQ1, respectively, all P for trend <0.001).

**Conclusions:**

Our results support that lower bone turnover biomarker (β-CTX, P1NP and OC) levels were associated with a combination of higher prevalence of insulin resistance and worse β-cell function among dysglycemia patients. It is feasible to detect bone turnover in diabetes or hyperglycemia patients to predict the risk of osteoporosis and fracture, relieve patients’ pain and reduce the expenses of long-term cure.

## Introduction

Bone metabolism and the blood glucose milieu are considered to be closely related (1). It is well recognized that diabetes or impaired glucose metabolism could affect bone health, contributing to decreased bone formation, increased bone marrow adiposity and increased risk of fracture (2, 3). In addition, there is a tight connection between bone metabolism and insulin resistance (IR) in type 2 diabetes (T2D) (4, 5). Insulin resistance may play a part in these interactions through impairment of the IGF-1 signaling pathway, which is vital for the muscle-bone unit (6). Homeostatic model assessment (HOMA) is a method for assessing IR and β-cell function from fasting glucose or insulin/C-peptide concentrations (7). The model has been used widely since it was first published in 1985 and has been included in numerous studies (8).

However, as the gold standard for the diagnosis of osteoporosis, bone mineral density (BMD) aberrations are not sufficient to predict the increased risk of osteoporosis and fracture for the hysteresis of imaging characteristics (9). Currently, bone turnover biomarkers are widely used to forecast the prevalence of bone metabolism dysfunction and estimate the treatment effect of osteoporosis (10–12). Thus, osteocalcin (OC), a protein secreted by osteoblasts, and the intact N-terminal propeptide of type I collagen (P1NP), all serves as a marker of bone formation. And β-C-terminal telopeptide (β-CTX) is regarded as a marker of bone resorption and osteoclast activity (10, 13, 14). In vivo research revealed that during the oral glucose tolerance test (OGTT), both formative (OC, P1NP) and resorptive (β-CTX) bone markers decreased within twenty minutes in healthy young individuals (15). However, a study also showed that serum levels of CTX (s-CTX) in postmenopausal, healthy, untreated women with T2D were not correlated with age, age at menopause, or BMI (16). This lack of correlation between s-CTX was also in accordance with the observations of Papakitsou et al. (17).

Research on the relationship between insulin resistance, glucose metabolism and bone turnover biomarkers in hyperglycemia patients remains scarce and inconclusive, thus clearly warranting further study. For this reason, the aim of the present study was to investigate the association of HOMA-IR and HOMA-%β with β-CTX, P1NP and OC in Chinese patients with hyperglycemia.

## Methods

### Study Design and Participants

**Figure 1:**
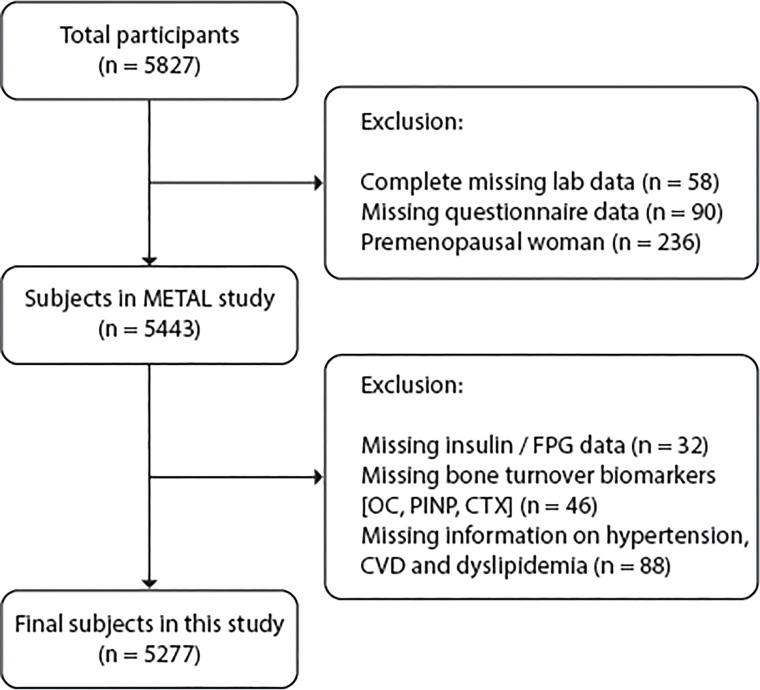
Flow chart of study participant selection (inclusions and exclusions).

We used data from volunteers in seven communities in Shanghai, China. The ongoing cross-sectional METAL study (Environmental Pollutant Exposure and Metabolic Diseases in Shanghai) is a population-based survey on the complications of metabolic diseases and risk factors (http://www.chictr.org.cn, ChiCTR1800017573). We included adults aged 18 years or older who had lived in their current area for more than six months and excluded subjects who were unwilling to participate or had severe communication problems or acute illness. Before data collection, all participants provided written informed consent. Our study initially enrolled 5827 populations aged 18 to 99 years old. Among them, subjects with missing laboratory results (n=58) or questionnaire data (n=90) and premenopausal women (n=236) were excluded. A woman was considered postmenopausal if she confirmed menopause on the questionnaire or was over 60 years old or over 55 years old with FSH ≥25 IU/L (18). Thus, 5443 participants were included in the METAL study. In this study, subjects without serum insulin and fasting plasma glucose (FPG) data (n=32), bone turnover data (n=46) and information on hypertension, cardiovascular disease (CVD) or dyslipidemia (n=88) were excluded. Thus, a total of 5277 subjects were ultimately involved in this study (**Figure 1**).

This study protocol was approved by the Ethics Committee of Shanghai Ninth People’s Hospital, Huangpu Branch of Shanghai Ninth People’s Hospital, Shanghai Jiaotong University School of Medicine. All following procedures were in accordance with the ethical standards of the responsible committee on human experimentation (institutional and national) and with the Helsinki Declaration of 1975.

### Data Collection

We followed the methods of Y. Wang et al. in 2019 (19). All data collection was performed by the same staff from the Department of Endocrinology and Metabolism in Shanghai Ninth People’s Hospital, Huangpu Branch of Shanghai Ninth People’s Hospital, Shanghai Jiaotong University School of Medicine who underwent a classic training program on the specific tools and methods used at every step of this study. A standard questionnaire was administered by trained staff to obtain information on demographic characteristics, self-reported symptoms, previous personal and family medical history, and risk elements in their daily lives. All experienced personnel were involved in the Survey on Prevalence in East China for Metabolic Diseases and Risk Factors (SPECT-China). In addition, weight, height, waist circumference and hip circumference were measured according to a standard protocol. All of the anthropometric measurements were conducted at the same time when the serum samples were collected.

### Clinical Measurements

We collected serum samples by venipuncture after an 8-hour fast from 6-10 o’clock in the morning. Within 2-4 hours, blood samples were stored at -20°C and shipped by air in dry ice to one central laboratory that was certified by the College of American Pathologists (CAP).

Insulin was detected by the chemiluminescence method (Abbott ARCHITECT i2000SR, Chicago, USA). FPG, glycosylated hemoglobin(HbA1c), total cholesterol (TC), triglycerides (TG), low-density lipoprotein (LDL) and high-density lipoprotein (HDL) were measured by a Beckman Coulter AU680 (Brea, USA).

Serum C-peptide was assessed by immunoassay (ARCHITECT i2000SR, Abbott Laboratories, Chicago, IL, USA), and 25(OH)D was detected using a chemiluminescence assay (ADVIA Centaur XP, Siemens, Germany).

Dysglycemia was consisted of three parts, T2DM, impaired fasting glucose (IFG) and impaired glucose tolerance (IGT). T2DM was determined using a previous diagnosis by health-care professionals, FPG level ≥ 7.0 mmol/L or HbA1c ≥ 6.5%. ADA_2003_ recommend cut points for IGT as 7.8–11.0 mmol/L measured at the 2-h time point of an OGTT. And IFG was defined as 5.6–6.9 mmol/L by ADA_2003_. Body mass index (BMI) was defined as weight (in kg) divided by height (in meters squared). In accordance with the Cooperative Meta-Analysis Group of the Working Group on Obesity in China criteria, BMI < 24 kg/m^2^ was considered normal, while BMI ≥ 24 kg/m^2^ was defined as overweight/obesity (20). Drug use was determined to take thiazolidinedione (TZD) drugs and insulin in the hypoglycemic therapy. Current smoking was defined as having at least 100 cigarettes over a lifetime and still smoking at present (21). Cardiovascular disease (CVD) was defined as a composite of a previous diagnosis of coronary heart disease, stroke or myocardial infarction, according to the self-reported record of the participants. Hypertension was defined as a systolic blood pressure of 140 mmHg or higher, a diastolic blood pressure of 90 mmHg or higher or a previous diagnosis by healthcare professionals. Dyslipidemia was diagnosed as a TC level≥ 6.22 mmol/L, triglycerides (TG) ≥ 2.26 mmol/L, LDL-C≥ 4.14 mmol/L, HDL-C< 1.04 mmol/L, or a self-reported physician’s diagnosis, as per the US modified National Cholesterol Education Program Adult Treatment Panel III guidelines (22).

### Outcome Definition

HOMA-IR has proven to be an instrumental tool for the assessment of insulin resistance and is the index of IR that is most widely applied in large population studies (23–25). The HOMA of IR and β-cell function was first described in 1985 (7, 8).

HOMA-IR and HOMA-%β are calculated using the following simplified equations:

HOMA-IR = (FPI×FPG)/22.5

HOMA-%β = (20×FPI)/(FPG - 3.5)

β-CTX, OC and P1NP were detected with a chemiluminescence method (Roche E602, Switzerland). The interassay coefficients of variation were as follows: 7.60% (β-CTX), 1.81% (OC) and 3.30% (P1NP). The intra-assay coefficients of variation were 5.50% (β-CTX), 0.80% (OC) and 3.0% (P1NP).

### Statistical Analyses

The survey analyses were performed with IBM SPSS Statistics, Version 22 (IBM Corporation, Armonk, NY, USA). A P value less than 0.05 was considered to be significant (two-sided). Continuous variables were expressed as the median (IQR, inter quartile range), and categorical variables were expressed as percentages (%). The nonparametric test and chi-square test were used to test for trends of variable changes across HOMA-IR and HOMA-%β quartiles, with the first quartile (Q1) representing the lowest quartile and the fourth quartile (Q4) being the highest, and to provide P-values that were adjusted for sex, age, BMI, lipids, vitamin D (VitD), C-peptide, current smoking and drinking habits, hypertension, CVD, dyslipidemia and drug use. For the association between HOMA and bone turnover biomarker levels, the model was adjusted for the same factors mentioned before. Multiple linear regression coefficients were applied to perform the statistical work.

## Results

### Clinical *C*haracteristics According to HOMA-IR and HOMA-%β Levels

**Table 1** presents the characteristics of the 5277 subjects with dysglycemia included in our study (n=5277). With increasing quartiles of HOMA-IR, the concentrations of CTX, P1NP and OC significantly decreased. In the HOMA-%β model, these three bone turnover markers significantly increased with β-cell function growth.

**Table 1:** Characteristics of subjects in terms of the quartiles (Q1-Q4) of HOMA-IR and HOMA-%β concentration.

Characteristics	HOMA-IR						
	Q1		Q2	Q3	Q4		P
	(<1.43)		(1.43-2.18)	(2.18-3.45)	(>3.45)		
Participants, n	1319		1319	1319	1320		―
Age, years	66(10)		67(10)	67(10)	67(10)		0.007
Men, %	50.72		45.56	44.05	40.76		<0.001
BMI	22.69(3.76)		24.21(3.8)	25.29(4.22)	26.11(4.97)		<0.001
Lipids	―		―	―	―		―
TC, mmol/L	5.22(1.46)		5.18(1.58)	5.06(1.59)	5.01(1.66)		<0.001
TG, mmol/L	1.18(0.75)		1.49(0.91)	1.68(1.1)	1.81(1.32)		<0.001
HDL-C, mmol/L	1.32(0.45)		1.21(0.38)	1.16(0.33)	1.1(0.34)		<0.001
LDL-C, mmol/L	3.17(1.04)		3.21(1.15)	3.17(1.13)	3.11(1.21)		0.011
VitD, ng/L	39.09(19.17)		38.73(18.09)	39.45(18.12)	38.45(16.69)		0.184
HbA1c,%	6.4(1.2)		6.7(1.3)	7.1(1.5)	7.8(2.07)		<0.001
C-peptide, ng/ml	1.08(0.4)		1.47(0.51)	1.83(0.69)	2.07(1.53)		<0.001
CTX, ng/ml	0.21(0.13)		0.2(0.12)	0.19(0.12)	0.18(0.12)		<0.001
P1NP, µg/L	42.59(24.37)		41.45(22.49)	39.99(21.47)	39.98(23.63)		<0.001
OC, ng/ml	12.5(6.3)		11.8(6.3)	10.8(5.6)	10.1(5.4)		<0.001
Drink, %	20.39		18.27	16.76	13.41		<0.001
Smoke, %	21.15		17.13	17.21	16.59		0.004
Hypertension, %	64.75		75.36	82.34	85.68		<0.001
CVD history, %	18.42		19.86	24.49	28.94		<0.001
Dyslipidemia, %	46.85		61.26	65.73	71.29		<0.001
Drug use,%	6.52		5.31	9.4	33.26		<0.001
Characteristics	HOMA-%β						
	Q1	Q2		Q3		Q4	P
	(<26.90)	(26.90-45.26)		(45.26-73.74)		(>73.74)	
Participants, n	1319	1319		1319		1320	―
Age, years	68(11)	67(10)		66(9)		66(10)	<0.001
Men, %	55.19	48.67		40.33		36.89	<0.001
BMI	23.5(4.2)	24.34(4.11)		24.67(4.46)		25.53(4.68)	<0.001
Lipids	―	―		―		―	―
TC, mmol/L	5.12(1.55)	5.08(1.51)		5.18(1.63)		5.09(1.62)	0.562
TG, mmol/L	1.4(1.06)	1.49(1.03)		1.56(1.04)		1.64(1.08)	<0.001
HDL-C, mmol/L	1.23(0.37)	1.18(0.39)		1.19(0.4)		1.17(0.36)	<0.001
LDL-C, mmol/L	3.17(1.09)	3.12(1.11)		3.22(1.17)		3.11(1.18)	0.486
VitD, ng/L	39.46(18.08)	39.19(18.2)		38.78(18.34)		38.4(17.68)	0.015
HbA1c,%	7.9(2.0)	7.1(1.4)		6.5(1.1)		6.4(1.2)	<0.001
C-peptide, ng/ml	1.22(0.59)	1.47(0.74)		1.63(0.91)		1.9(1.11)	<0.001
CTX, ng/ml	0.18(0.12)	0.19(0.13)		0.21(0.13)		0.21(0.13)	<0.001
P1NP, µg/L	36.19(18.37)	40.17(21.56)		42.84(23.49)		45.68(25.07)	<0.001
OC, ng/ml	10.3(5)	11.3(5.6)		12(6.35)		11.9(6.53)	<0.001
Drink, %	21.38	17.89		15.31		14.24	<0.001
Smoke, %	22.52	19.18		16.38		14.02	<0.001
Hypertension, %	73.84	78.39		76.57		79.32	0.005
CVD history, %	19.48	23.81		22.9		25.53	0.001
Dyslipidemia, %	56.33	58		64.52		66.29	<0.001
Drug use,%	10.92	12.05		10.16		21.36	<0.001

Data are expressed as the median (IQR) for continuous variables and as percentages for categorical variables. The nonparametric test and chi-square test were used.

In both the HOMA-IR and HOMA-%β models, BMI, TG, C-peptide and prevalence of hypertension, CVD and dyslipidemia were positively related to the increasing concentration quartile (P for trend <0.001, **Table 1**).

### Association of HOMA-IR With Bone Markers in Diabetes Patients

**Figure 2:**
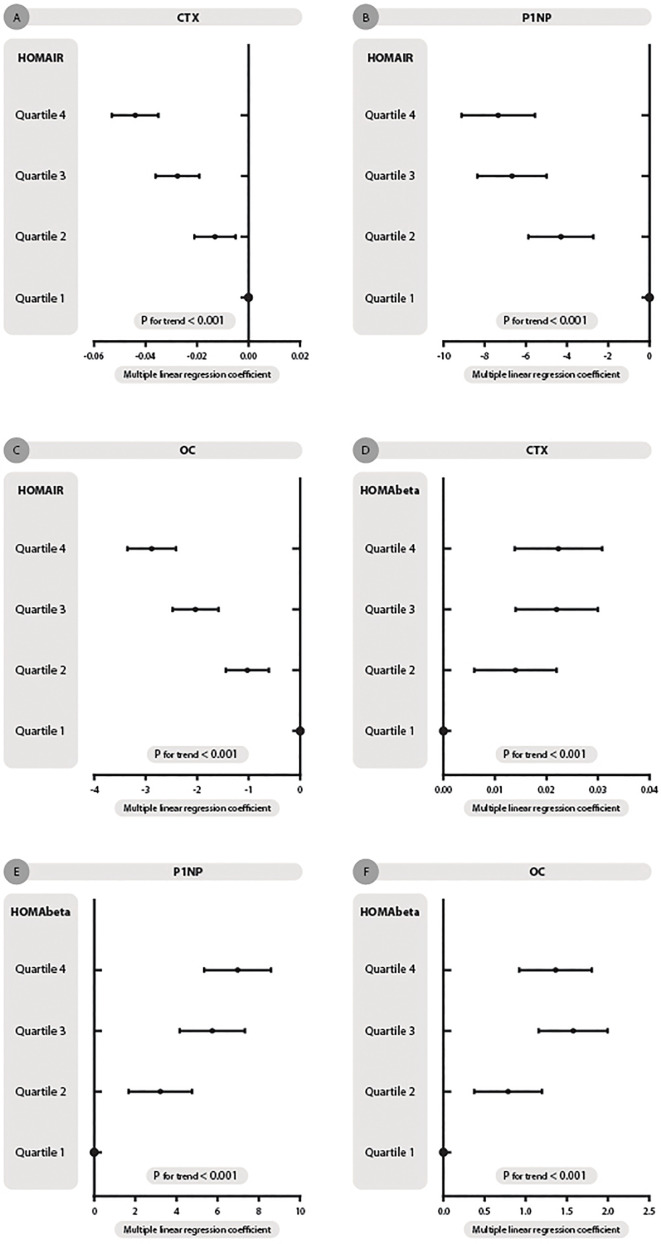
HOMA-IR and HOMA-beta quartiles with changes in the association of β-CTX, P1NP and OC concentration in dysglycemia patients. **(A–C)** represent HOMA-IR quartiles with β-CTX, P1NP and OC concentration trends, respectively. **(D–F)** represent HOMA-beta quartiles with three turnovers mentioned. Multiple linear regression coefficients were adjusted for sex, age, BMI, lipids, Vit D, C-peptide, current smoking and drinking, hypertension, CVD, dyslipidemia and drug use.

The association of HOMA-IR concentration and bone markers in the diabetes and hyperglycemia populations is presented in **Figure 2** and **Table 2**. HOMA-IR concentration was negatively related to CTX, and as HOMA-IR quartiles increased, the level of CTX decreased (P< 0.001, **Table 2**) after adjusting for sex, age, BMI, lipids, VitD, C-peptide, current smoking and drinking, hypertension, CVD, dyslipidemia and drug use. [Q1: regression coefficient (β): -0.015, 95% CI: -0.018, -0.012; Q2: β -0.013, 95% CI: -0.021, -0.005; Q3: β -0.028, 95% CI: -0.036, -0.019; Q4: β -0.044, 95% CI: -0.053, -0.035; p for trend < 0.001; **Figure 2A**].

**Table 2:** The description table for **Figure 2**.

Bone turnover	HOMA-IR							P for trend
B (95% CI)	Q1	Q2		Q3			Q4	
CTX, ng/ml	-0.014(-0.018, -0.012)	-0.013(-0.021, -0.005)		-0.028(-0.036, -0.019)			-0.044(-0.053, -0.035)	<0.001
PINP, µg/L	-2.40(-2.98, -1.83)	-4.30(-5.88, -2.72)		-6.67(-8.35, -5.00)			-7.34(-9.13, -5.55)	<0.001
OC, ng/ml	-0.97(-1.12, -0.81)	-1.03(-1.44, -0.61)		-2.04(-2.48, -1.59)			-2.89(-3.36, -2.41)	<0.001
Bone turnover	HOMA-%β							P for trend
B (95% CI)	Q1	Q2		Q3			Q4	
CTX, ng/ml	0.007(0.005, 0.010)	0.014(0.006, 0.022)		0.022(0.014, 0.030)			0.022(0.014, 0.031)	<0.001
PINP, µg/L	2.34(1.81, 2.86)	3.21(1.67, 4.76)		5.73(4.16, 7.31)			6.95(5.30, 8.60)	<0.001
OC, ng/ml	0.49(0.35, 0.63)	0.79(0.38, 1.20)		1.58(1.16, 2.00)			1.36(0.92, 1.80)	<0.001

The description form of **Figure 2** represents the association of HOMA-IR and HOMA-beta quartiles and β-CTX, P1NP and OC concentrations. The multiple linear regression coefficient was adjusted for the same correlative factors as in **Figure 2**.

Thus, HOMA-IR was also negatively related to P1NP after adjusting for the same factors mentioned above [Q1: β -2.404, 95% CI: -2.978, -1.830; Q2: β -4.298, 95% CI: -5.878, -2.718; Q3:β -6.673, 95% CI: -8.351, -4.996; Q4: β -7.340, 95% CI: -9.130, -5.550; p for trend < 0.001; **Figure 2B**].

As displayed in **Table 2** and **Figure 2C**, after full adjustment, HOMA-IR also had a negative relationship with OC [Q1: β -0.965, 95% CI: -1.116, -0.813; Q2: β -1.025, 95% CI: -1.442, -0.609; Q3:β -2.036, 95% CI: -2.478, -1.593; Q4: β -2.885, 95% CI: -3.357, -2.412; p for trend < 0.001; **Figure 2C**].

### Association of HOMA Beta With Bone Markers in Diabetes Patients

As shown in **Table 2**, HOMA-%β had a positive relationship with the three bone markers after adjusting for sex, age, BMI, lipids, VitD, C-peptide, current smoking and drinking, hypertension, CVD, dyslipidemia and drug use.

CTX concentration increased when HOMA-%β quartiles increased [Q1: β 0.008, 95% CI: 0.005, 0.010; Q2: β 0.014, 95% CI: 0.006, 0.022; Q3:β 0.022, 95% CI: 0.014, 0.030; Q4: β 0.022, 95% CI: 0.014, 0.031; p for trend < 0.001. **Figure 2D**].

Thus, P1NP level had the same variation trend as HOMA-%β concentration [Q1: β 2.338, 95% CI: 1.813, 2.862; Q2: β 3.213, 95% CI: 1.671, 4.756; Q3:β 5.733, 95% CI: 4.156, 7.310; Q4: β 6.951, 95% CI: 5.300, 8.602; p for trend < 0.001. **Figure 2E**].

HOMA-%β concentration also had a positive relationship with OC [Q1: β 0.488, 95% CI: 0.348, 0.628; Q2: β 0.786, 95% CI: 0.376, 1.197; Q3:β 1.575, 95% CI: 1.155, 1.995; Q4: β 1.361, 95% CI: 0.921, 1.800; p for trend < 0.001. **Figure 2F**].

## Discussion

Bone turnover is a product of the tightly coupled processes of bone formation and resorption, with the net balance between the two determining the bone mass and serum calcium level. This process requires the input of numerous hormones (such as parathormone, calcitonin and VitD), growth factors (growth hormone, IGF–1) and cytokines that interact at the level of osteoclasts and osteoblasts to regulate the balance between net resorption and formation (26). In this study, three typical biomarkers were selected for evaluation of the relationship between insulin resistance and pancreatic β-cell function in dysglycemia patients.

In this cross-sectional study, we reported the association of bone metabolism with insulin resistance and β-cell function in diabetic and hyperglycemic populations. The results suggest that β-CTX, P1NP and OC were negatively associated with HOMA-IR and positively associated with HOMA-%β. These results indicated that patients with higher bone metabolism have a lower prevalence of insulin resistance and better β-cell function. To the best of our knowledge, this is the first study to estimate the bone metabolism state in dysglycemia patients and emphasize the association of bone turnover biomarkers with insulin resistance and β-cell function in an investigation with a large community-dwelling sample.

Insulin signaling is an evolutionarily conserved pathway that plays a pivotal role in the regulation of metabolism and longevity, and bone is an insulin-responsive organ. In T2D, impaired insulin signaling in peripheral tissues leads to insulin resistance (27). Thus, metabolic disturbances associated with diabetes increase the risk of fragility fractures along with increased bone marrow adiposity. Bone marrow adipose tissue (BMAT) accounts for approximately 8% of the total fat mass, representing a significant fat accumulation site in adult humans. BMAT participates in regulating whole body energy metabolism through its ability to respond to insulin (28), activate Sirt1, which is a key cellular energy sensor, and induce a thermogenic gene program (29). In addition, BMAT also responds to insulin-sensitizing antidiabetic medications such as TZD drugs and PPARγ agonists (28, 30, 31). Therefore, when insulin resistance occurs, BMAT fills the interstitial bone, the microarchitecture deteriorates, and the structure of the trabecular bone undergoes early changes prior to the BMD decrease. This means that subclinical osteoporosis could happen without the detection of a low T-score for the lag effect. An early efficient method for determining bone loss is needed to facilitate preventive treatment among the population with insulin resistance.

The clinical usefulness of bone turnover biomarkers in the contemporary management of osteoporosis can be described as follows. On the one hand, it has been suggested that several of these markers can be used to target populations at increased risk for osteoporosis, assess the treatment effect of calcium supplements and predict the development of future fractures (32–34). On the other hand, their role in estimating BMD or bone loss in an individual patient has been revealed (32). Thus, there could exist an inner link for bone biomarkers to estimate the relationship between the process of osteoporosis and the function of pancreatic or insulin resistance. Previous research held the opinion that insulin resistance had a negative relationship with bone formation and a positive interrelation with bone resorption and that pancreatic function had the opposite relationship (35, 36). However, our results from this study showed that when IR was aggravated or β-cell function decreased, the metabolic disorders of bone agitated not only formation but also resorption. This would be due to the increasing requirements of the components of bone formation, and the process of bone resorption also accelerates to provide the materials to maintain the dynamic balance.

Currently, with the continuous aging of the global population and changes in modern lifestyle, type 2 diabetes mellitus and osteoporosis have become public health issues that always coexist. A recent study indicated an increased risk of brittle bone fracture as another concerning complication of diabetes (37). In addition to the most common complications, such as macrovascular disease, nephropathy, retinopathy, and peripheral neuropathy (38), osteoporotic fractures have a considerable impact on individual health as well as on the cost to society since they lead to a significant increase in not only overall mortality but also long-term morbidity and major disabilities (39, 40). Up to a point, it might be feasible to detect bone turnover biomarkers in dysglycemia patients with insulin resistance or with low-level β-cell function to predict the risk of osteoporosis along with BMD determination and the hazard of fracture. The dynamic excellence of biomarkers could be detected after a month of supplementation with therapy, and clinical intervention would perform better according to early feedback (40). Physicians could predict the curative effect within 3-6 months. Therefore, identifying glycemia patients at high risk for future fractures is significant in order to develop effective preventive treatment to reduce occurrence, relieve patients’ pain and lessen the expenses of long-term cure.

Finally, this study has several limitations. First, our study population came from seven communities in Shanghai, China. The results do not represent other regions in China or other ethnic groups. Second, the findings suggest a relationship between insulin resistance and bone metabolism without indicating causation; further prospective follow-up studies are needed. Third, it would be more convincing to detect bone mineral density along with bone turnover biomarkers to elucidate the progress of osteoporosis and the development of preventive treatment.

## Conclusion

Insulin resistance and β-cell function were significantly associated with bone turnover in this cross-sectional study of a large population of dysglycemia participants. The three bone biomarkers decreased when insulin resistance was aggravated. When pancreatic β-cell function advanced, CTX, P1NP and OC were all elevated in combination. It might be feasible to detect bone turnover in abnormal glucose metabolism patients with insulin resistance or with low-level β-cell function to predict the risk of prevalence of osteoporosis along with determining BMD and the hazard of fracture. Therefore, it is significant to identify these populations at high risk for future fractures to develop effective preventive treatment to reduce occurrence, relieve patients’ pain and lessen the expenses of long-term cure.

## Data Availability Statement

The raw data supporting the conclusions of this article will be made available by the authors, without undue reservation.

## Ethics Statement

The studies involving human participants were reviewed and approved by the Ethics Committee of Shanghai Ninth People’s Hospital, Huangpu Branch of Shanghai Ninth People’s Hospital, Shanghai Jiaotong University School of Medicine.

## Author Contributions

YL and ZA contributed to the conception and design of the study. HG and CW contributed to acquisition, analysis, and interpretation of data. HG, CW, BJ, SG, JC, YiZ, RY, KZ, and JZ drafted the article. NW, CZ, CC, LZ, TG, and YaZ critically revised the manuscript for important intellectual content. All authors contributed to the article and approved the submitted version.

## Funding

This study was supported by National Natural Science Foundation of China (91857117); Science and Technology Commission of Shanghai Municipality (19140902400, 18410722300); the Major Science and Technology Innovation Program of Shanghai Municipal Education Commission (2019-01-07-00-01-E00059); Commission of Health and Family Planning of Pudong District (PWZxq2017-17); Municipal Human Resources Development Program for Outstanding Young Talents in Medical and Health Sciences in Shanghai (2017YQ053); Shanghai JiaoTong University School of Medicine (19XJ11007). The funders played no role in the design or conduct of the study, collection, management, analysis, or interpretation of data or in the preparation, review, or approval of the article.

## Conflict of Interest

The authors declare that the research was conducted in the absence of any commercial or financial relationships that could be construed as a potential conflict of interest.

## Abbreviations

T2DM, type 2 diabetes mellitus; IFG, impaired fasting glucose; IGT, impaired glucose tolerance; HbA1c, glycosylated hemoglobin A1c; IR, insulin resistance; HOMA, homeostatic model assessment; β-CTX, β-C-terminal telopeptide; P1NP, intact N -terminal propeptide of type I collagen; OC, osteocalcin; BMD, bone mineral density; BMI, body mass index; OGTT, oral glucose tolerance test; TC, total cholesterol; TG, triglycerides; HDL-C, high-density lipoprotein cholesterol; LDL-C, low-density lipoprotein cholesterol; VitD, Vitamin D; FPG, fasting plasma glucose; IGF-1, insulin-like growth factor-1; CVD, cardiovascular disease; CI, confidence interval; SD, standard deviation; BMAT, bone marrow adipose tissue; TZD, thiazolidinedione.
